# 2712. Respiratory syncytial virus (RSV) pre-fusion F antibody titers in hematopoietic cell transplant recipients with upper versus lower respiratory tract infections

**DOI:** 10.1093/ofid/ofad500.2323

**Published:** 2023-11-27

**Authors:** Sara R Kim, Elizabeth M Krantz, Claire Andrews, Khaleel Yahya, Terry L Stevens-Ayers, Chikara Ogimi, Janet A Englund, Emily T Martin, Michael J Boeckh, Alpana Waghmare

**Affiliations:** Seattle Children's Hospital, Seattle, Washington; Fred Hutch Cancer Center, Seattle, Washington; Fred Hutchinson Cancer Center, Seattle, Washington; Fred Hutchinson Cancer Center, Seattle, Washington; Fred Hutchinson Cancer Center, Seattle, Washington; National Center for Child Health and Development, Setagaya-ku, Tokyo, Japan; Seattle Children’s Hospital, Seattle, Washington; University of Michigan, Ann Arbor, MI; Fred Hutchinson Cancer Center, Seattle, Washington; Seattle Children's Hospital/Fred Hutchinson Cancer Center, Seattle, Washington

## Abstract

**Background:**

Humoral immune responses to respiratory syncytial virus (RSV) infection in hematopoietic cell transplant (HCT) recipients have not been well characterized. We measured RSV pre-fusion F antibody (RSVpreF) titers in HCT recipients in 3 groups: upper respiratory tract infections (URTI) only, lower respiratory tract infections (LRTI) only, and those who progressed from URTI to LRTIs (progressors).

**Methods:**

We evaluated adult and pediatric allogeneic HCT recipients who acquired RSV from 12/2011 to 12/2019. Prospective and leftover clinical serum specimens were collected from the Fred Hutchinson Cancer Center. Samples included baseline prior to infection, at RSV URTI or LRTI diagnosis, and after infection (both 4-6 and >8 weeks). Quantitative RSVpreF titers were determined using the MesoScale Discovery electrochemiluminescence assay. We compared longitudinal log_10_ RSVpreF titers among groups using linear mixed effect models with restricted cubic splines.

**Results:**

A total of 129 samples from 39 patients were analyzed; 24 with URTI only, 8 progressors, and 7 with LRTI only. The median age at infection was 45 years, but HCT recipients with LRTI only had an overall higher median age of 54 (range 23-63) (Table 1). Most had acute leukemia (n=18, 46%) and received peripheral blood stem cells (n=27, 69%) as their cell source (Table 1). Median RSVpreF values were similar across the 3 different groups at each timepoint (Table 2). Trajectories of RSVpreF titers over time for each patient trended towards an increase following RSV diagnosis in all groups (Figure 1a). Predicted curves in RSVpreF titers over time did not differ significantly by group (Figure 1b, p=0.38).

**Figure:**
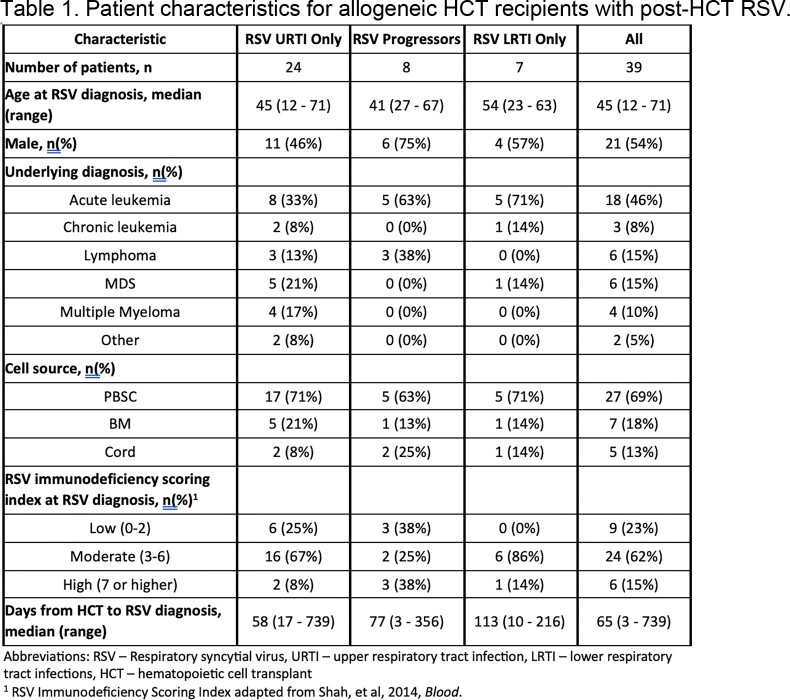

**Table 2.**
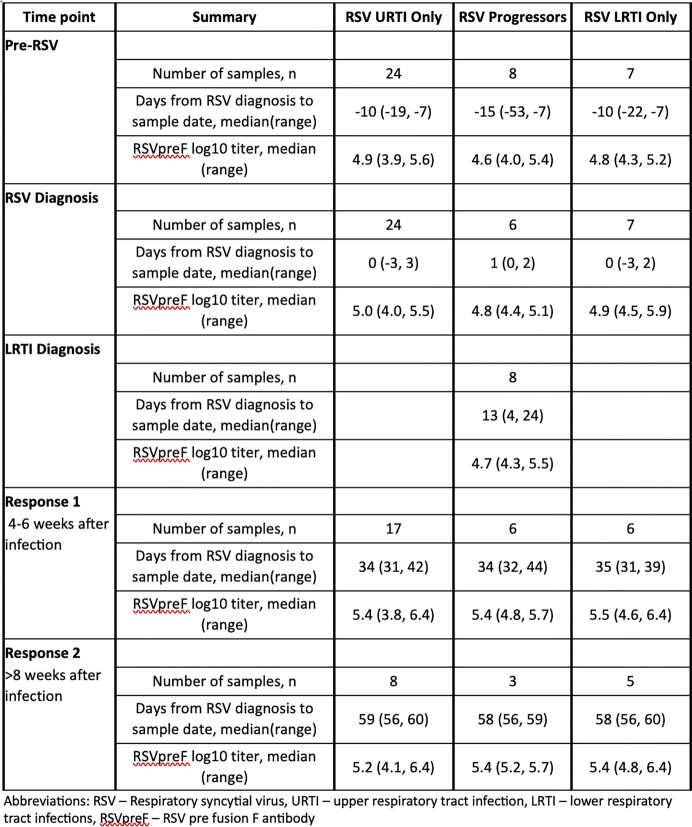
Summary of RSVpreF titers among allogeneic transplant recipients before and after a post-HCT RSV infection. RSVpreF titers were obtained utilizing Meso Scale Discovery chemiluminescence assays.

**Figure:**
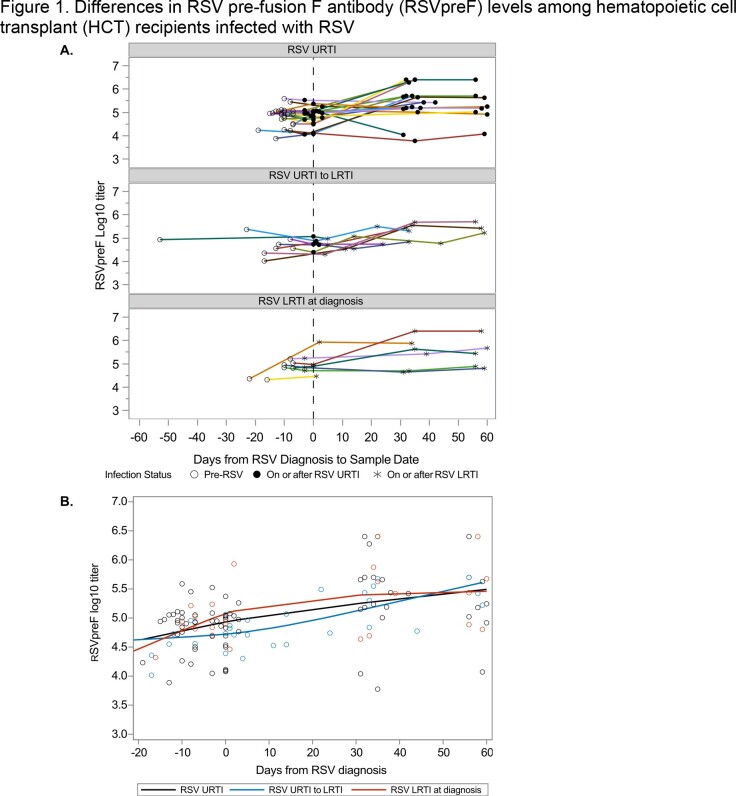

(A) RSVpreF titers for allogeneic HCT recipients with RSV infection. Each line represents a patient. The top panel shows patients with RSV URTI only, the middle panel shows patients who progressed from URTI to LRTI, and the bottom panel shows patients who presented with RSV LRTI. Open circles represent time points prior to RSV diagnosis, filled circles represent time points just prior to, on or after URTI, and asterisks represent time points just prior to, on, or after LRTI. (B) RSVpreF titers plotted by days from RSV diagnosis. Open circles represent observed values and lines represent model predicted trajectories. Black circles and lines correspond to patients with RSV URTI only, blue corresponds to patients who progressed from RSV URTI to LRTI, and dark orange represents patient who presented with RSV LRTI at diagnosis. An outlying observation at day -53 was excluded.

**Conclusion:**

Among allogeneic HCT recipients with RSV infection, RSVpreF titers increased in response to RSV infection, but did not differ among patients with URTI only, progressors, or those with LRTI only across pre and post infection time points. Our novel immunoassay results are similar to those from previously described RSV neutralization assays, but extended the observation period beyond 4 weeks after infection, revealing a modest increase in RSVpreF titers. Further characterization of RSV humoral immunity, including mucosal immunity, is needed.

**Disclosures:**

**chikara Ogimi, MD**, bioMerieux Japan Ltd.: Honoraria|Horiba: Honoraria|Pfizer: Honoraria **Janet A. Englund, MD**, Ark Biopharma: Advisor/Consultant|AstraZeneca: Advisor/Consultant|AstraZeneca: Grant/Research Support|GlaxoSmithKline: Grant/Research Support|Meissa Vaccines: Advisor/Consultant|Merck: Grant/Research Support|Moderna: Advisor/Consultant|Moderna: Grant/Research Support|Pfizer: Advisor/Consultant|Pfizer: Grant/Research Support|Sanofi Pasteur: Advisor/Consultant **Emily T. Martin, PhD, MPH**, Merck: Grant/Research Support **Michael J. Boeckh, MD PhD**, Allovir: Advisor/Consultant|Amazon: Grant/Research Support|Ansun: Grant/Research Support|Merck: Advisor/Consultant|Merck: Grant/Research Support|Moderna: Advisor/Consultant|Symbio: Advisor/Consultant **Alpana Waghmare, MD**, Allovir: Grant/Research Support|Amazon: Grant/Research Support|Ansun Biopharma: Grant/Research Support|GlaxoKlineSmith/Vir: Grant/Research Support|Kyorin Pharmaceuticals: Advisor/Consultant|Pfizer: Grant/Research Support|Vir Biotechnology: Advisor/Consultant

